# RUNX1 (RUNX family transcription factor 1), a target of microRNA miR-128-3p, promotes temozolomide resistance in glioblastoma multiform by upregulating multidrug resistance-associated protein 1 (MRP1)

**DOI:** 10.1080/21655979.2021.2009976

**Published:** 2021-12-11

**Authors:** Jianglong Xu, Jia Song, Menglin Xiao, Changsheng Wang, Qisong Zhang, Xiaoye Yuan, Shaohui Tian

**Affiliations:** a Department of Neurosurgery, Affiliated Hospital of Hebei University, Baoding, China; b School of Basic Medicine, Hebei University, Baoding, China

**Keywords:** Glioblastoma multiform, temozolomide, RUNX1, miR-128-3p

## Abstract

Glioblastoma multiform (GBM) is the most frequent type of malignant brain tumor with a poor prognosis. After optimal surgery, radiotherapy plus temozolomide (TMZ) is the standard treatment for GBM patients. However, the development of TMZ resistance limits its efficacy in GBM management. Runt Related Transcription Factor 1 (RUNX1) and microRNAs have been implicated in drug resistance of TMZ in GBM. In this study, we revealed the underlying mechanism of TMZ resistance and identified miR-128-3p/RUNX1 axis as a novel target for TMZ resistance in GBM. RUNX1 expression was significantly upregulated in GBM tissues as compared to normal tissues, and its expression was even higher in recurrent GBM tissues and TMZ-resistant GBM cells. RUNX1 depletion inhibited the viability, proliferation, migration, invasion and TMZ resistance of GBM cells, which could be rescued by RUNX1 overexpression. We further identified miR-128-3p as a tumor-suppressor whose overexpression restored the sensitivity of TMZ in GBM cells. miR-128-3p negatively regulated RUNX1 and subsequently downregulated multidrug resistance-associated protein 1 (MRP1). Together, the present study indicates that RUNX1 confers TMZ resistance in GBM by upregulating MRP1, which is negatively regulated by miR-128-3p. Targeting miR-128-3p/RUNX1/MRP1 axis provides a potential strategy to overcome TMZ resistance in GBM.

## Introduction

Glioblastoma multiform (GBM) is the highest-grade form of glioma, as well as the commonest invasive and malignant brain tumor in both adult and pediatric patients, which has a remarkably poor prognosis [1–3]. Although tremendous efforts have been made in the study of GBM, the etiology and pathophysiology are largely unclear. Current standard treatment for newly diagnosed GBM combines surgery, radiotherapy and adjuvant chemotherapy with temozolomide (TMZ) [3]. TMZ is the first line clinical drug, however, most patients develop resistance to TMZ therapy when GBM recurs [4–6]. The acquisition of TMZ resistance is a major impediment for GBM therapy, accounting for the majority of fatality [7]. Due to the aggressive nature of GBM and the prevalence of drug resistance, virtually all patients with GBM suffer from cancer relapse and the median overall survival is dismally low [8,9]. For this reason, managing recurrent GBM is extremely challenging due to the lack of other effective therapeutic agents. Understanding the detailed molecular mechanisms underlying TMZ resistance could provide insight into novel strategy development to improve the treatment outcome in GBM.

Runt Related Transcription Factor 1 (RUNX1) has been recognized as an indispensable transcription factor for hematopoiesis. It belongs to the RUNX family of transcriptional and post-transcriptional modulators characterized of a highly conserved runt domain [10,11]. In tumors of the central nervous system, RUNX1 expression is significantly higher in Mesenchymal (Mes) subtype of GBM and closely related to Mes subtype initiation through microRNA (miR)-mediated mechanisms [12]. A previous study indicated that RUNX1 contributes to malignant behavior of GBM [13]. Recently, a study further reported that RUNX1 overexpression can dramatically promote proliferation and invasion of GBM [14]. It has also been reported that the downregulation of RUNX1 restores TMZ sensitivity and inhibits glioma progression [15]. However, the regulatory mechanisms of RUNX1 expression in GBM remains enigmatic.

MicroRNAs (miRNAs) are a class of endogenous non-coding RNAs with about 22 nucleotides in length, which are capable of regulating the translation or the degradation of target mRNAs via binding to the 3′-untranslated regions (3′-UTR) [16]. MicroRNAs are implicated in multiple cell functions in cancers, including GBM [17–21]. Of note, many studies have shown that the dysregulation of miRNAs is strongly associated with the development of TMZ resistance in GBM [22–25]. Whether miRNAs contribute to RUNX1-dependent regulation of TMZ resistance in GBM remains to be elucidated.

Multidrug resistance-associated protein 1 (MRP1) belongs to the MRP superfamily of transporters [26]. Accumulating evidence has shown that MRP1 is a modulator of cellular chemoresistance, which is overexpressed in brain tumors [27], and TMZ has been recognized as a substrate of MRP1 [28]. The mechanism underlying MRP1 upregulation in GBM is largely unknown.

This study investigated the underlying mechanism of TMZ resistance and identified miR-128-3p/RUNX1 axis as a novel target for TMZ resistance in GBM. We validated the oncogenic role of RUNX1 in GBM cells. Based on miRNA databases (Targetscan, miRTarbase and miRWalk), we identified miR-128-3p as a negative regulator of RUNX1. We further showed that MRP1 was upregulated by RUNX1 to confer TMZ-resistance. These results collectively indicate that miR-128-3p/RUNX1/MRP1 axis regulates TMZ-resistance in GBM cells, which could serve as potential targets to modulate TMZ-sensitivity in GBM.

## Methods

### Cell culture and treatment

U87MG and A172 human glioblastoma cell lines were obtained from the China Infrastructure of Cell Line Resource. C8-DA astrocyte cell line was purchased from ATCC (Maryland, USA). All cells were cultured in Dulbecco’s-modified Eagle’s medium (DMEM) (HyClone, USA) containing 10% FBS (FBS, Gibco, USA), 100 IU/ml penicillin (GIBCO, U.K.), at 37°C in a humidified incubator. Cells were sub-cultured every three days. TMZ-resistant U87MG and A172 cells were established by culturing the cells in 100 µM TMZ for 4 weeks.

### Cell viability assay analysis

Cell viability was evaluated with the trypan blue staining and CCK-8 assay kit (Solarbio, Beijing, China) in 96-well culture plates as previously described [29]. For trypan blue staining, cell suspension in 1 × 10^6^ cells/mL was mixed with 0.4% trypan blue solution at a ratio of 9:1 for 20-min incubation at 37°C. Dead stained with blue color was photographed and counted under microscope. The percentage of cell viability was calculated by the formula: cell viability (%) = (the number of alive cells/the number of total cells) ×100%. For CCK-8 assay, U87MG and A172 cells were seeded in to a 96 -well plate at a density of 1500 cell/well and cultured in a humidified cell culture incubator for 0, 24, 48, 72 and 96 hours, respectively. CCK-8 solution (10 μL) was added to each well at indicated time point for 2h incubation. The light absorption value (OD value) in each condition was captured at 450 nm on a Synergy H1 microplate reader (Winooski, Vermont, USA).

### Plasmid construction and cell transfection

The plasmids containing the sequence of RUNX1 cDNA (pcDNA-RUNX1), miR-128-3p mimic and inhibitor were synthesized by Hanbio Biotechnology (Shanghai, China). Small interfering RNA (siRNA) of RUNX1 and the negative control si-NC was purchased from GenePharma (Shanghai, China). SiRNA, miRNA mimic, and plasmid were transfected in U87MG and A172 cells using Lipofectamine 2000 (Invitrogen Carlsbad, CA, USA) according to the manufacturer’s instruction.

Briefly, cells were seeded in 6-well plates at a density of 5 × 10^5 cells/well. 24 hours later, indicated quantity of molecules or 6 µg of plasmid was added into 100 µl Opti-MEM® I Reduced-Serum Medium (Invitrogen, Carlsbad, CA, USA), and then 6 µL Lipofectamine 2000 reagent was added for 10 min incubation at room temperature. The mixture was added into the cell culture dropwise. 48 h after transfection, cells were harvested for further experiments.

### Apoptosis

The percentage of apoptotic cells was detected as previously described [30]. U87MG and A172 cells were trypsinized and resuspended in Annexin V binding buffer (FITC-Annexin V Apoptosis Detection Kit with 7-AAD, Biolegend, California, USA) at a concentration of 1.0 × 10^6^ cells/mL. 5 μL Annexin V-FITC and 5 μL 7-AAD reagent were added to 1 mL cell suspension of 1 million cells, and incubated for 30 mins in the dark. Stained cells were refilled with 400 μL Annexin V binding buffer. The percentage of apoptotic cells was detected by BD FACS CantoTM II Flow Cytometer (BD Biosciences).

### Colony formation assay

Colony formation assay was performed as previously described [31]. U87MG and A172 cells with different transfection treatment were inoculated on six-well plates at the density of 1000 cells/well and cultured for 14 days, and the culture medium was changed every 3 days during the period. After 14 days, cells were fixed with 4% paraformaldehyde at room temperature for 10 mins and stained with Giemsa reagent 0.5% crystal violet (Beyotime, Shanghai, China) for 20 mins. Subsequently, the number of colonies was counted under Leica AM6000 microscope (Leica, Wetzlar, Germany).

### Transwell migration and invasion assay

Transwell assay was performed as previously described [32]. Cells with different treatments were trypsinized and resuspended in serum-free medium. The transwell upper chamber (Corning, New York, USA) without Matrigel (BD Biosciences, Bedford, MA, USA) was used for migration assay, while transwell chamber coated with Matrigel was used for invasion assay. Cells with different treatments were trypsinized and resuspended in serum-free medium at a density of 2 × 105 cells/mL. 100 µL cell suspension was inoculated into transwell upper chamber and 500 μL of 10% serum-containing medium was added to the lower chamber. After 24 hours, cells were fixed with 10% methanol for 10 min and stained with crystal violet (Beyotime, Shanghai, China) for 20 min at room temperature. Stained cells were counted under a light microscope (Olympus, Tokyo, Japan).

### Dual luciferase reporter assay

Luciferase reporter assay was performed as previously described [32]. The sequence containing the wild type binding site between miR-128-3p and RUNX13ʹUTR or the sequence with mutated binding site was cloned into the PmirGLO vector expressing firefly luciferase respectively (Promega, Madison, WI, USA). The reporter plasmid and Renilla luciferase (hRlucneo) control plasmid were co-transfected into cells in the presence of either miR-128-3p mimic or miR-NC in a 12-well plate (1 × 10^5 cells/well) using Lipofectamine 2000 reagent. 48 h post transfection, the relative luciferase activities were measured using Dual-Luciferase Reporter Assay Kit (Promega, Madison, WI, USA) on the Fluoroskan Ascent FL microplate reader (Thermofisher Scientific, USA).

### RNA fluorescence in situ hybridization (FISH)

Specific probes for miR-128-3p (Cy3 fluorophore) and RUNX1 (Cy5 fluorophore), and a control probe (Cy5 fluorophore) were synthesized by (RiboBio, Guangzhou, China) and detected using a fluorescence in situ hybridization kit (RiboBio, Guangzhou, China) followed by the manufacturer’s instructions. Cells seeded on the cover slip were fixed with 4% paraformaldehyde and permeabilized with 0.05% Triton X-100. 50 nM miR-128-3p probe and RUNX1 probe, or miR-128-3p probe and control probe were added to the cells in hybridization buffer for 2-hour incubation. Cells was washed with TBST buffer for three times and then placed on the slide with mounting media containing DAPI (Vector Lab, Inc., Burlingame, CA, United States). Finally, the intensity and localization of each probe were observed by confocal laser microscope (Nikon, Japan) and the Pearson’s Correlation Coefficients for colocalization was analyzed by Image J software (Bethesda, MD, USA).

### Databases

Three databases were utilized to predict miRNA targets of RUNX1, including miRTarbase (http://mirtarbase.cuhk.edu.cn/php/index.php), TargetScan 5.1 (http://www.targetscan.org/), and the miRWalk target prediction server (http://www.mirwalk.umm.uni-heidelberg.de/). Gene Expression Profiling Interactive Analysis (GEPIA: http://gepia.cancer-pku.cn/index.html) was used to analyze the expression level of RUNX1 in GBM. The Cancer Genome Atlas (TCGA: https://www.cancer.gov/about-nci/organization/ccg/research/structural-genomics/tcga) was used to analyze the relationship between RUNX1 expression and the overall survival of GBM patients.

### Western blot analysis

Western blot analysis was conducted following previous description [33]. Cells were lysed for 30 min in cold RIPA Lysis Buffer (Beyotime, Shanghai, CN) containing protease inhibitor cocktail (Thermofisher Scientific, Waltham, MA, USA). The lysate was centrifuged at 16,000 rpm for 15 mins at 4°C, and the supernatant was collected. Protein concertation in the supernatant was quantified by a BCA Protein assay kit (Beyotime, Shanghai, China). 10 µg protein sample was mixed with loading buffer and denatured at 99°C for 5 mins, and then used for SDS-PAGE electrophoresis. Separated protein in SDS-PAGE gel was transferred onto the PVDF membrane (BioRad, CA, USA). After blocking with 5% skimmed milk for 1 hour, the membrane was incubated with primary antibodies overnight: anti-RUNX1 (1:1000, Affinity, China), anti-MRP1 (1:1000, CST, USA), and anti-β-actin (1:5000, GeneTex, USA). The membrane was washed 3 times with TBST for 5 minutes each. After wash, the membrane was further incubated with HRP-linked secondary antibody (1:3000; CST, USA) at room temperature for 1 hour. After wash, the protein bands were developed using an ECL kit (Solarbio, Beijing, China) and photographed using a gel imager system (Bio-Rad, CA, USA). The densitometry analysis was performed with Image J software (Bethesda, MD, USA).

### Patients and tissue samples

To investigate the expression of RUNX1 between primary and recurrent GBM, primary and recurrent GBM tumor samples were collected from 15 patients by surgical resection from patients diagnosed with GBM in the Affiliated Hospital of Hebei University. All the patients were subject to optimal surgery and radiochemotherpay after primary diagnosis. When the patients were diagnosed with GBM relapse, second surgery was performed to collect the recurrent GBM tumor samples. All experimental ethics were approved by the Institutional Ethics Committee at Affiliated Hospital of Hebei University. The clinicopathological feature of the enrolled patients were summarized in the Table 1.

**Table 1.** Clinicopathological feature of the enrolled patients

Characteristics	Patients (%) (n = 15)
**Age (median)**	25–76 (42.0)
**Gender**	
Male	9 (60.0)
Female	6 (40.0)
**WHO grade**	
I	2 (13.3)
II	6 (40.0)
III	4 (26.6)
IV	3 (20.0)
**Histological type**	
Astrocytomas	11 (73.3)
Oligodendrogliomas	3 (20.0)
Ependymomas	1 (6.7)

### Quantitative RT-PCR (qRT-PCR)

Quantitative RT-PCR was performed as previously described [33]. Total RNA was extracted using TRIzol Reagent (Invitrogen, CA, USA) as per the instruction. 1 µg of total RNA was converted into cDNA using PrimeScript RT kit (Takara, Tokyo, Japan). qPCR was conducted for gene expression analysis using SYBR Green® Premix Ex Taq (Takara). Relative gene expression was calculated based on threshold cycle (Ct) values and normalized against β-actin expression using comparative 2^−ΔΔCT^ method. Primers were listed as follows: RUNX1 upstream: CCTCCGGTAGTAATAAAGGCTTCTG, downstream: CCGATTGAG-TAAGGACCCTGAA. miR-128-3p upstream: CTGGTAGGTCACAGTGAACCG, downstream: TCAACTGGTGTCGTGGAGTC. GAPDH upstream: GTTGTCTCCTGCGACTTCA, downstream: GGTGGTCCAGGGTTTCTTA.

### Immunohistochemistry

Histological analyses of RUNX1 protein in tumor tissues were performed with the avidin-biotin-peroxidase method as previously described [34]. Briefly, the tumor tissues embedded in paraffin was cut into 4-μm-thick sections and were deparaffinized and rehydrated. After antigen unmasking by heating in citrate solution (SignalStain® Citrate Unmasking Solution (10X), CST, USA) for 15 mins, the sections were washed in dH2O three times and incubated in 3% hydrogen peroxide for 10 min. After three times washes in TBST buffer, the section was blocked 1 hour in 5% normal goat serum, and then incubated with primary anti-RUNX1 antibody (1:1000, Affinity, China) overnight with shaking. The section was washed three times using TBST buffer and soaked in 3 drops of SignalStain® Boost Detection Reagent (HRP-Rabbit, CST, USA) for 30 min at room temperature. Then 200 µL SignalStain® substrate (CST, USA) was added to each section for 5 minutes. The section was washed in dH2O two times and mounted with coverslips using the mounting medium (CST, USA). The images were photographed under a confocal microscope (Nikon, Japan).

### Statistical analysis

Statistical tests were performed using GraphPad Prism software 7.0 (Version7.00, U.S.A.). All data are expressed as mean ± SEM. Differences between percentages were evaluated by Fisher’s exact test. Comparison of continuous variables between two groups were assessed using unpaired two-tailed Student t test, and the comparison above three groups were analyzed using one-way ANOVA followed by Tukey or Dunnett tests. A Kaplan–Meier test was used to estimate the overall survival from lifetime data of the patients and the log-rank test was used to examine differences in survival. *P* value lower than 0.05 (*P* < 0.05) was considered statistically significant.

## Results

This study investigated the role of RUNX1 in GBM and identified the functional interaction between miR-128-3p and RUNX1, and validated that RUNX1-dependent MRP1 expression mediated TMZ sensitivity in GBM. RUNX1 expression was significantly higher in GBM tissues as compared to normal tissues, and its upregulation was also observed in recurrent GBM tissues and TMZ-resistant GBM cells. RUNX1 depletion suppressed the proliferation, migration, invasion and TMZ resistance of GBM cells. We further demonstrated that miR-128-3p functioned as a tumor-suppressor whose overexpression restored TMZ sensitivity in GBM cells. miR-128-3p downregulated RUNX1 and subsequently reduced the expression of multidrug MRP1. All the results indicated that miR-128-3p/RUNX1/MRP1 axis is implicated in TMZ sensitivity in GBM cells, which could serve as potential targets to modulate TMZ resistance in GBM.

### RUNX1 expression is upregulated in recurrent GBM patients

**Figure 1.**
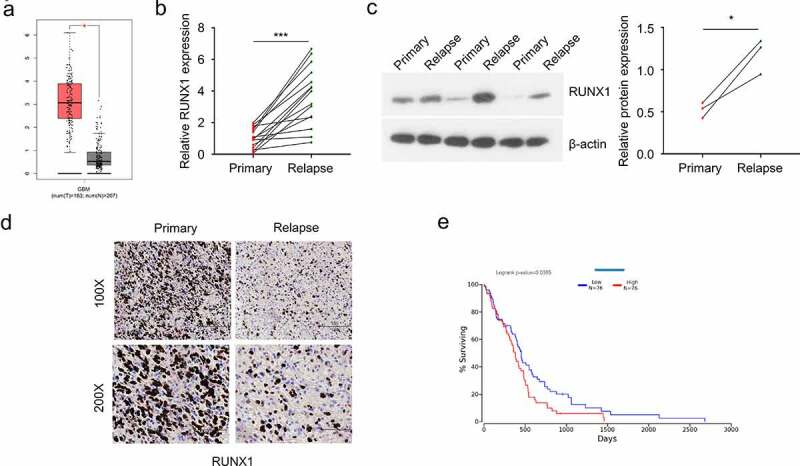
RUNX1 is highly expressed in GBM and associated with a poor prognosis (a) The expression of RUNX1 in GBM tumors and normal brain tissues was compared using data from GEPIA database. (b-d) Relative mRNA and protein level of RUNX1 in primary and recurrent GBM samples, analyzed by qRT-PCR (b), Western blot (c) and immunochemistry (d) E. Kaplan–Meier survival assessment of GBM patients, which were divided into low and high RUNX1 expression groups (n = 76 in each group, P < 0.05). Each sample was analyzed in 3 independent experiments. * *P* < 0.05 and *** *P* < 0.001

To analyze the expression level of RUNX1 in GBM, we first used GEPIA tool and found that RUNX1 was significantly upregulated in GBM tumor samples as compared to the normal brain tissues (Figure 1a). Besides, primary and recurrent GBM samples from 15 GBM patients were collected. qRT-PCR, Western blot and immunohistochemistry were performed to assess the mRNA and protein levels of RUNX1. mRNA level and protein levels of RUNX1 in recurrent GBM samples were significantly higher than that in primary tumors (Figure 1b-d). We also retrieved the survival data of 152 GBM patients from TCGA database and divided them into high expression group (n = 76) and low expression group (n = 76) according to the median RUNX1 expression. The overall survival (OS) of patients in high expression group was significantly lower than the low expression group (Figure 1e), which indicates that high RUNX1 expression is associated with poor prognosis of GBM patients.

### RUNX1 regulates TMZ sensitivity in GBM cells

**Figure 2.**
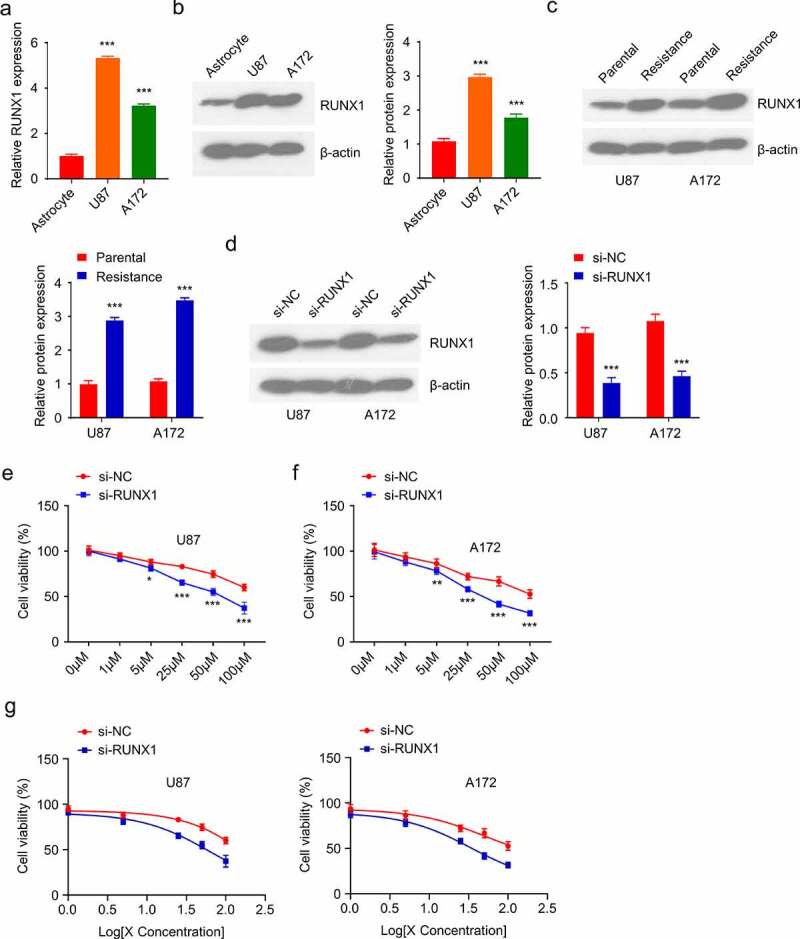
RUNX1 is required for TMZ resistance in GBM cells (a-b) mRNA and protein levels of RUNX1 in U87 and A172 GBM cell lines as compared to astrocytes C8-DA. (c) Western blot analysis of RUNX1 in TMZ-sensitive parental cells and TMZ-resistant cells. (d) The level of RUNX1 in U87 and A172 cells was analyzed after being transfected with RUNX1-siRNA and control siRNA (si-NC). (e-g) Cell viability (e-f) and IC50 (g) of RUNX1-silenced cells was compared to the cells treated with si-NC. Cells were treated with different TMZ concentration (0, 1 μM, 5μM, 25 μM, 50 μM, 100 μM, 24 h), and trypan blue assay was performed to quantify the viable cells. Each sample was repeated in at least 3 independent experiments. * *P* < 0.05, ** *P *< 0.01, and *** *P* < 0.001

To further investigate the intonement of RUNX1 in regulating TMZ resistance of GBM, we first compared the level of RUNX1 in U87 and A172 glioblastoma cell lines to C8-DA astrocyte cells. Both the mRNA and protein expression levels of RUNX1 were markedly higher in U87 and A172 cells when compared to C8-DA cells (Figure 2a,b). In addition, TMZ-resistant U87 and A172 cell lines were established by culturing the cells in medium containing 100 µM TMZ for 4 weeks. As compared to the parental cells, RUNX1 protein level was significantly upregulated in TMZ-resistant cells (Figure 2c). We further evaluated the functional requirement of RUNX1 in TMZ-resistance by transfecting the resistant cells with RUNX1-siRNA. Western blot revealed the reduced level of RUNX1 after the knockdown by siRNA (Figure 2d). Trypan blue assay showed that TMZ knockdown decreased the cell viability in both U87 and A172 cell lines at different concentrations and as well as the IC50 of TMZ treatments (Figure 2e,f,g). Together, these data showed that RUNX1 is required for TMZ resistance in GBM cells.

### RUNX1 knockdown inhibits cell proliferation, migration and invasion

**Figure 3.**
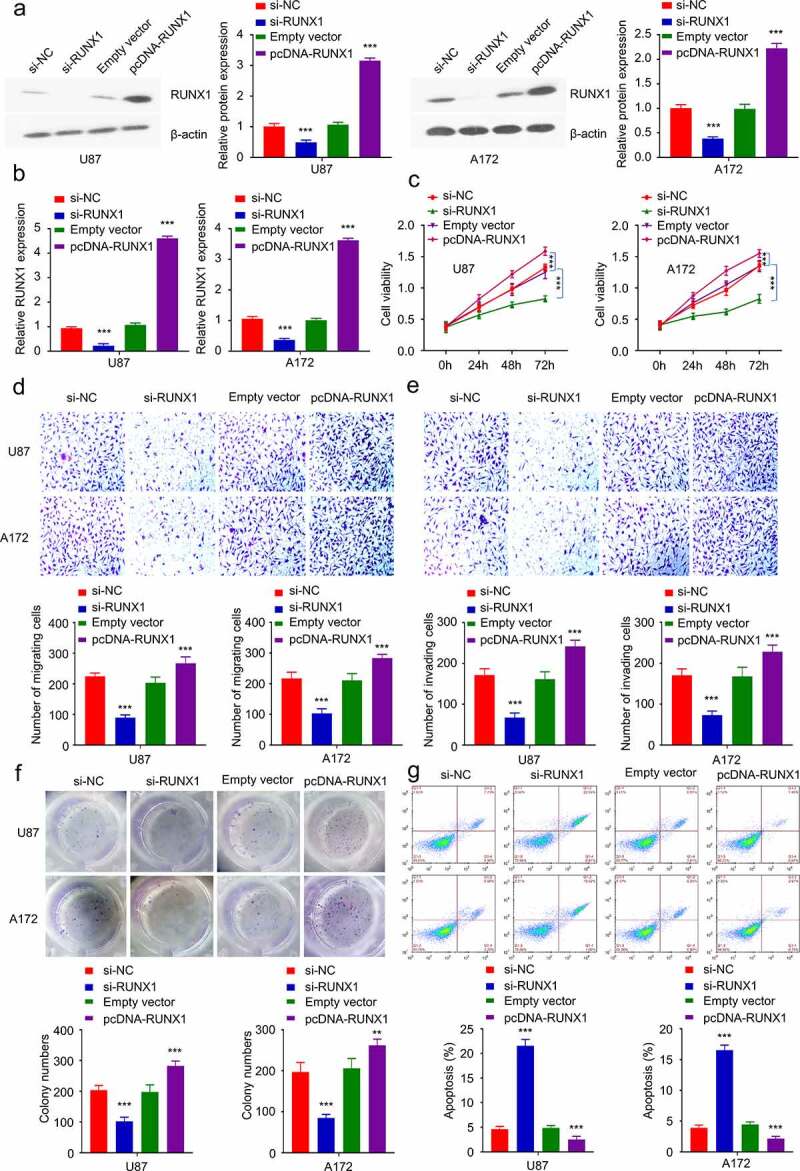
RUNX1 regulates tumor cell proliferation, migration and invasion. (a-b) The knockdown or overexpression of RUNX1 after the transfection with si-RUNX1 or pcDNA-RUNX1 expression vector was analyzed by Western blot and qRT-PCR in U87 and A172 cells. (c) CCK-8 assay in cells after transfection with si-NC, si-RUNX1, Empty vector or pcDNA-RUNX1. (d) Transwell migration assay, (e) Transwell invasion assay, (f) colony formation assay, (g) apoptosis were performed in U87 and A172 cells after transfection with si-NC, si-RUNX1, Empty vector or pcDNA-RUNX1. Each sample was repeated at least 3 times. * *P* < 0.05, ** *P *< 0.01, and *** *P* < 0.001

To validate the functional role of RUNX1 in GBM cells, we performed loss-of-function and gain-of-function experiment by either silencing RUNX1 (si-RUNX1) or overexpressing RUNX1 (pcDNA-RUNX1 expression plasmid) in both U87 and A172 cell lines (Figure 3a,b). As revealed by CCK-8 proliferation assay, silencing RUNX1 suppressed cell proliferation while the overexpression of RUNX1 significantly promoted cell proliferation. (Figure 3c). Cell migration/invasion assay further demonstrated that silencing RUNX1 impaired cell migration and invasion, while RUNX1 overexpression promoted cell migration (Figure 2d,e). Furthermore, downregulation of RUNX1 markedly impaired colony formation ability and induced cell apoptosis, and RUNX1 overexpression enhanced colony formation and prevented cell apoptosis (Figure 2f,g).

### RUNX1 is negatively targeted by miR-128-3p in GBM cells

**Figure 4.**
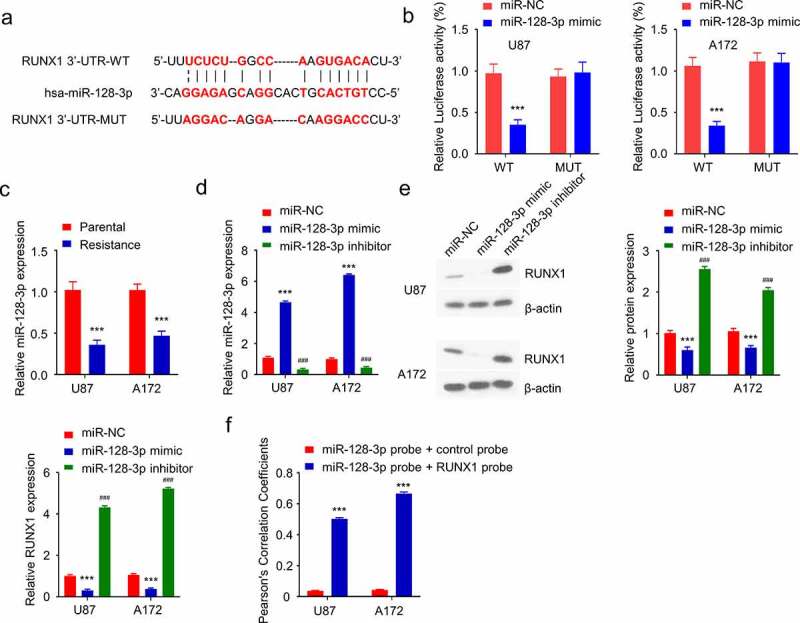
RUNX1 is a direct target of miR-128-3p. (a) Alignment of the miR-28-3p sequence with that of RUNX1 3ʹ-UTR. (b) Dual luciferase reporter assay was performed in U87 and A172 cells in the presence of miR-NC or miR-128-3p. WT: wildtype reporter containing binding site. MUT: reporter with mutated binding site. (c) The expression of miR-28-3p in TMZ-sensitive parental cells and TMZ-resistant cells. (d) Expression level of miR-28-3p in TMZ-resistant cells after transfection with miR-128-3p mimic or miR-128-3p inhibitor. (e) Gene and protein expression levels of RUNX1 in TMZ-resistant cells transfected miR-128-3p mimic or miR-128-3p inhibitor. (f) Fluorescence in situ hybridization (FISH) was performed using miR-128-3p probe + control probe or miR-128-3p probe + RUNX1 probe to investigate the co-localization of miR-128-3p and RUNX1 in GBM cell lines. Each sample was repeated at least 3 times. *** *P* < 0.001

We next sought to search for potential regulatory miRNAs targeting RUNX1. miR-128-3p was identified as a potential target which showed a binding site in the 3ʹUTR region of RUNX1 using three miRNA prediction databases (Targetscan, miRTarbase and miRWalk) (Figure 4a). Dual luciferase reporter assay was performed in both U87 and A172 cell lines using WT reporter containing wildtype binding site as well as the MUT reporter with mutated binding site. The presence of miR-128-3p mimic significantly suppressed the luciferase activity of the WT reporter, which was not observed in the MUT reporter (Figure 4b), indicating that miR-128-3p negatively regulates RUNX1 mRNA. Next, the level of miR-128-3p in parental and TMZ-resistant cell lines was examined by qRT-PCR, and miR-128-3p was significantly downregulated in TMZ-resistant cells (Figure 4c). To validate the regulatory role of miR-128-3p on RUNX1 expression, miR-128-3p mimic and inhibitor were transfected into TMZ-resistant cells, which increased or decreased the level of miR-128-3p respectively (Figure 4d). qRT-PCR and Western blot showed that RUNX1 was downregulated by miR-128-3p mimic while miR-128-3p inhibitor increased Runx1 expression (Figure 4e). Furthermore, fluorescence in situ hybridization (FISH) analysis revealed that there was significant colocalization of miR-128-3p and RUNX1 mRNA in GBM cell lines. (Figure 4f). Together, these results indicate that miR-128-3p is a negative regulator of RUNX1 expression.

### MiR-128-3p attenuates TMZ resistance in GBM cells by downregulating RUNX1

**Figure 5.**
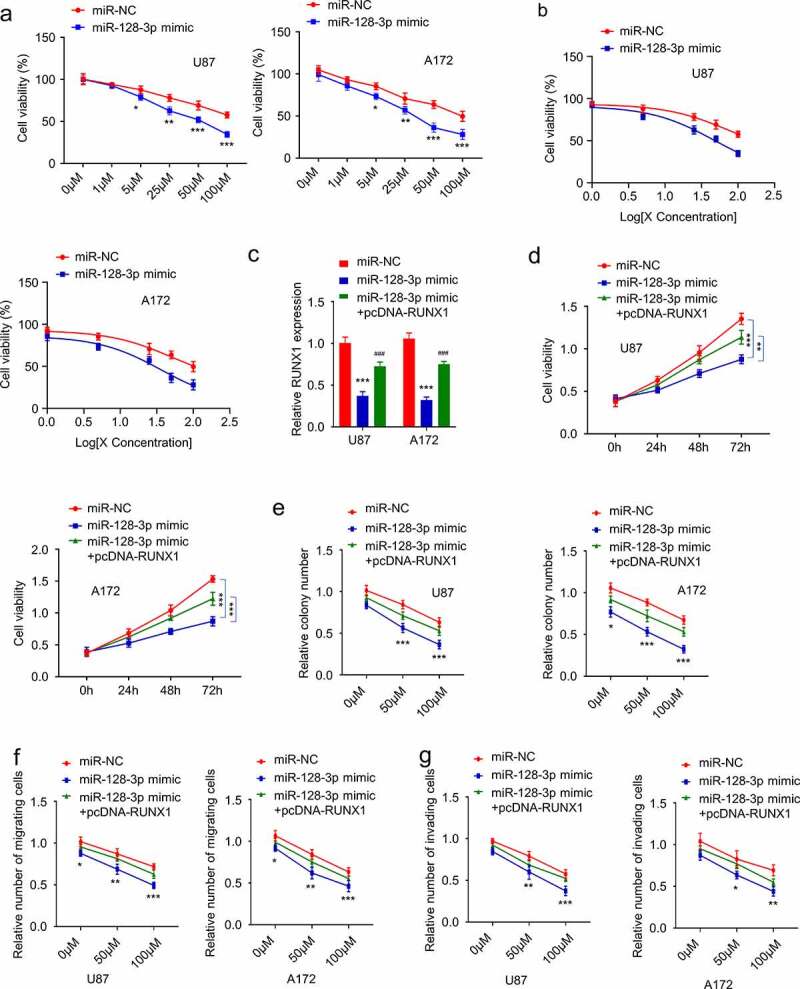
MiR-128-3p attenuates TMZ resistance in GBM by targeting RUNX1 (a-b) Cell viability and IC50 of cells transfected with miR-128-3p mimic or miR-NC in the increasing concentration of TMZ treatment (0, 1 μM, 5 μM, 25 μM, 50 μM, 100 μM, 24 h). (c) qRT-PCR analysis of RUNX1 expression in U87 and A172 cells transfected with miR-NC, miR-128-3p mimic and miR-128-3p mimic + pcDNA-RUNX1. (d) Cell viability of U87 and A172 cells transfected with miR-NC, miR-128-3p mimic and miR-128-3p mimic + pcDNA-RUNX1 after TMZ treatment (50 μM) for 0 h, 24 h, 48 h and 72 h. (e) Colony formation assay, (f) Transwell migration assay, (g) Transwell invasion assay were performed in U87 and A172 cells transfected with miR-NC, miR-128-3p mimic and miR-128-3p mimic + pcDNA-RUNX1 after TMZ treatment (0, 50 μM, 100 μM). Each experiment was repeated at least 3 times. * *P* < 0.05, ** *P *< 0.01, and *** *P* < 0.001

To demonstrate the functional involvement of miR-128-3p in TMZ resistance, we treated U87 and A172 cells with miR-128-3p mimic, and then the cells were subject to with different concentrations of TMZ treatment for 24 hours. Trypan blue assay showed that miR-128-3p mimic sensitized the cells to TMZ treatment and lowered the IC50 (Figure 5a and b). We further investigated whether the effect of miR-128-3p could be rescued by RUNX1 overexpression. Cells were transfected with miR-NC, miR-128-3p mimic or miR-128-3p mimic + pcDNA3.1-RUNX1. qRT-PCR analysis showed that RUNX1 expression reduced by miR-128-3p mimic, which could be partially restored by pcDNA3.1-RUNX1 (Figure 5c). Cells in different groups were treated with TMZ (50 μM) for different durations and CCK-8 assay revealed that miR-128-3p mimic significantly increased the sensitivity to TMZ and RUNX1 overexpression partially restored the resistance (Figure 5d). Similar results were obtained using colony formation and transwell assay. Overexpression of miR-128-3p markedly inhibited the proliferation, migration and invasion abilities of cells, while overexpression of RUNX1 impaired the effects of miR-128-3p to a certain extent (Figure 5d,e,f).

### RUNX1/miR-128-3p axis regulates MRP1 expression in TMZ-resistant GBM cells

**Figure 6.**
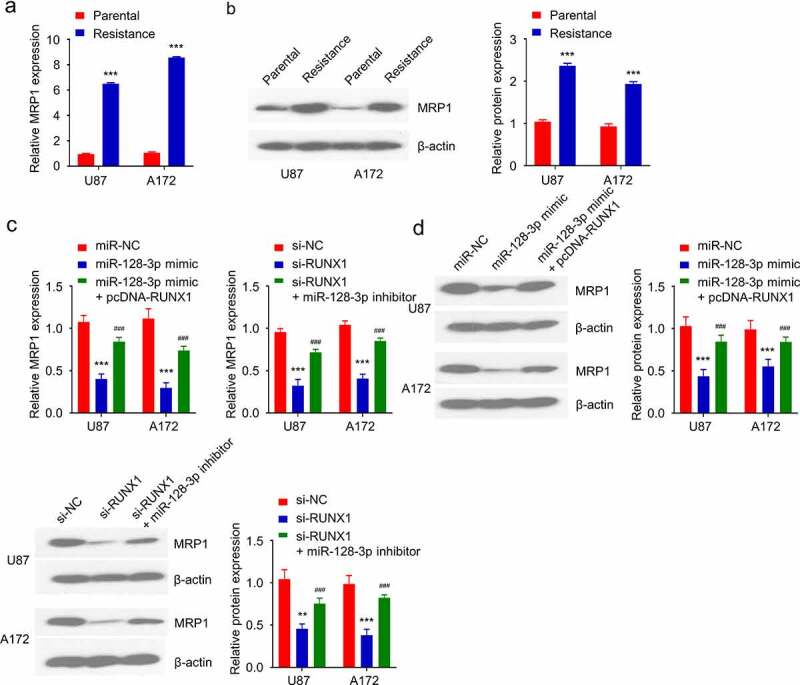
RUNX1/miR-128-3p axis regulates MRP1 expression in TMZ-resistant GBM cells. (a-b) Gene and protein level of MRP1 in TMZ-sensitive parental cells and TMZ-resistant cells were detected by qRT-PCR and Western blot. (c-d) Gene and protein levels of MRP1 were examined in cells transfected with miR-NC, miR-128-3p mimic, miR-128-3p mimic+ pcDNA-RUNX1 or si-NC, si-RUNX1 and si-RUNX1+ miR-128-3p inhibitor. Each sample was repeated at least 3 times. *** *P* < 0.001

Multidrug resistance is usually accompanied by the increased expression of MRP1 [35]. As expected, the mRNA and protein levels of MRP1 in TMZ-resistant cells were significantly higher than that in the parental cells (Figure 6a,b). In order to explore whether RUNX1/ miR-128-3p axis regulates MRP1 expression, cells were transfected with miR-128-3p mimic, miR-128-3p mimic+ pcDNA-RUNX1 or RUNX1-siRNA and RUNX1-siRNA miR-128-3p inhibitor. These results showed that miR-128-3p overexpression downregulated MRP1, which was rescued by RUNX1 overexpression (Figure 6c,d). Similarly, silencing RUNX1 significantly reduced MRP1 level, which could be rescued by miR-128-3p inhibitor (Figure 6c,d). These results demonstrated that RUNX1/miR-128-3p axis regulates MRP1 expression in GBM cells.

## Discussion

GBM is a progressive debilitating cancer in the central nervous system (CNS) and takes up over 50% of brain-related carcinomas in adults [36], which is associated with a very poor prognosis [29,37]. Despite the application of combined radiotherapy and chemotherapy with TMZ after optimal surgery, most patients eventually develop TMZ resistance and suffer from the malignant relapse of aggressive tumors [38–40].Recent studies revealed that RUNX1 and miRNAs are implicated in the development of resistant phenotype of TMZ in GBM [41–43]. Here, the present study identified miR-128-3p as a negative regulator of RUNX1 and validated their functional interaction in TMZ sensitivity of GBM cells, suggesting that miR-128-3p/RUNX1 axis could serve as a potential therapeutic target in TMZ-resistant GBM.

In CNS carcinoma, RUNX1 upregulation was reported in the Mes state of GBM. A study based on a context-specific gene regulatory network showed that RUNX1 mediates the expression of Mes signatures and is linked to the unfavorable prognosis in GBM patients GBM through an extensive microRNAs network [12,44–46]. Through the analysis of patient data in GEPIA, this study verified that RUNX1 is highly expressed in GBM tumor tissues. Their aberrant upregulation is also observed in the recurrent GBM tumors, suggesting a potential role of RUNX1 overexpression to GBM relapse. High expression of RUNX1 is associated with poor prognosis in GBM patients, indicating that RUNX1 overexpression is an unfavorable factor in GBM patients. Furthermore, the selection of TMZ-resistant cells fosters the upregulation of RUNX1, and RUNX1 upregulation confers TMZ-resistance in GBM cells. RUNX1 upregulation also promotes the malignant phenotype such as cell proliferation, migration and invasion. Together, our study highlighted the oncogenic role of RUNX1 in GBM.

Owing to the versatile roles in a wide spectrum of biological processes, miRNAs can function as oncogenes or tumor-suppressors by regulating different targets. Recently the dysregulation of miRNAs was reported in a variety of malignancies, which was proposed as potential biomarkers or therapeutic targets [47–50]. Particularly, miRNAs were reported to regulate multiple phenotypes of GBM, such as cell proliferation, migration and invasion, self-renewal, angiogenesis and drug resistance [51–53]. Our study identified a novel mechanism by which miR-128-3p binds to the 3ʹUTR region of RUNX1 and downregulates its expression. Overexpression of miR-128-3p re-sensitizes GBM cells to TMZ treatment by downregulating RUNX1. Therefore, our data highlighted the regulatory role of miR-128-3p in TMZ sensitivity.

MRP1, a member of MRP transporter family, was found to be upregulated in various tumors to mediate drug resistance [54–56]. Accumulating evidence has showed that increased expression of MRP1 is responsible for TMZ drug resistance in high grade of GBM [57,58]. Inhibiting MRP1 is proposed as an attractive approach to improve treatment for recurrent GBM [15,59–61]. However, the regulation of MRP1 expression in GBM is largely unclear. In the present study, we provided evidence that miR-128-3p/RUNX1 axis regulates MRP1 expression in GBM cells. RUNX1 silencing or miR-128-3p overexpression reduces MRP1 expression in TMZ-resistant cells, suggesting that MRP1 is a potential target of RUNX1 to modulate TMZ sensitivity GBM.

## Conclusion

In summary, our study identified the functional interaction between miR-128-3p and RUNX1 in the regulation of TMZ resistance of GBM cells, and validated the role of RUNX1-dependent MRP1 expression in TMZ sensitivity in GBM. However, the functional role of miR-128-3p and RUNX1 in TMZ sensitivity needs to be further validated in animal model. Furthermore, the expression pattern of miR-128-3p in GBM samples has not been established. Future work will focus on GBM patient samples to investigate the expression pattern of miR-128-3p, its diagnostic and prognostic values as well as the transcriptional regulation.
